# MetalDock: An Open Access Docking Tool for Easy and Reproducible Docking of Metal Complexes

**DOI:** 10.1021/acs.jcim.3c01582

**Published:** 2023-12-04

**Authors:** Matthijs L. A. Hakkennes, Francesco Buda, Sylvestre Bonnet

**Affiliations:** † Leiden Institute of Chemistry, Leiden University, P.O. Box 9502, 2300 RA Leiden, The Netherlands

## Abstract

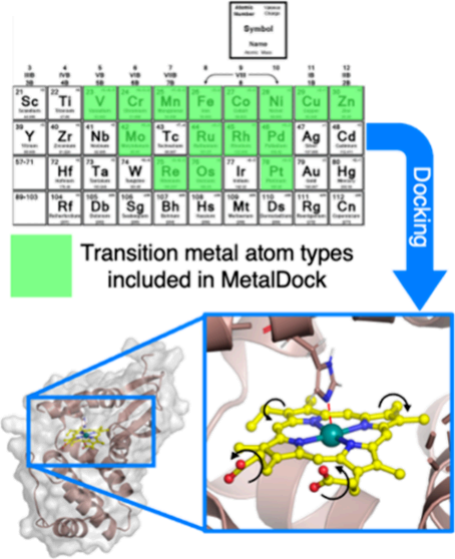

Despite the proven potential of metal complexes as therapeutics, the lack of computational tools available for the high-throughput screening of their interactions with proteins is a limiting factor toward clinical developments. To address this challenge, we introduce MetalDock, an easy-to-use, open access docking software for docking metal complexes to proteins. Our tool integrates the AutoDock docking engine with three well-known quantum software packages to automate the docking of metal–organic complexes to proteins. We used a Monte Carlo sampling scheme to obtain the missing Lennard-Jones parameters for 12 metal atom types and demonstrated that these parameters generalize exceptionally well. Our results show that the poses obtained by MetalDock are highly accurate, as they predict the binding geometries experimentally determined by crystal structures with high spatial reproducibility. Three different case studies are presented that demonstrate the versatility of MetalDock for the docking of diverse metal–organic compounds to different biomacromolecules, including nucleic acids.

## Introduction

Metal–organic compounds have been shown to be a versatile class of molecules that can be used as reagents or catalysts.^1,2^ However, they also show the ability to interact with biomolecules such as proteins or DNA.^3^ The latter property can be of special interest for the development of new bioactive molecules such as anticancer (pro)drugs.^4−8^ The introduction of a metal atom into a small-molecule drug can lead to biological activity via two interaction pathways. First, the metal center may be coordinatively saturated, in which case it is often thought of as a sort of hypervalent carbon atom directing the organic functional groups of its ligands toward the protein interior. Octahedral square-planar metal complexes may thereby extend the chemical space available for the development of small-molecule drugs, next to purely organic compounds.^4,9−13^ Alternatively, the metal atom has one (or several) vacancy in its first coordination sphere and may directly interact with the protein backbone via coordination of metal-binding residues such as histidine, methionine, cysteine, or aspartic acid.^14,15^ Such unsaturated metal complexes are then close to organic “suicide inhibitors” that can form (partially) covalent bonds with serine or cysteine residues of the catalytic center of the protein. A priori, these two types of interactions with proteins are rich enough to open numerous opportunities for the rapid development of metal–organic compounds as protein-targeted drugs.

However, the lack of available software to perform easy docking of metal compounds to known proteins has, up to now, slowed the clinical developments of these compounds. In fact, one of the main obstacles in developing metallodrugs is the limited number of computational tools for in silico metallodrug screening. As the electronic structure of metals is best described with quantum mechanics, a quantum mechanical/molecular mechanical (QM/MM) simulation would in principle be required for the most accurate description of the interaction between a metal complex and a protein.^16^ Unfortunately, this level of theory is not suitable for high-throughput screening (HTS), as the sum of simulation times for thousands of compounds would be intractable even for the current state-of-the-art supercomputers. Hence, concessions need to be made, and a switch from quantum mechanics to classical mechanics is required.

In HTS protocols, the initial step in the analysis of a compound typically involves performing a molecular docking simulation to predict the most energetically favorable conformation of the compound when it is bound to its target protein. The procedure starts by defining a box around a specific site of interest (typically the substrate-binding center of an enzyme), after which the compound is translated and rotated, and its torsional degrees of freedom are sampled. Each conformation is then evaluated by a semiempirical energy function, from which the most probable structure can be deduced based on a predefined scoring function. For organic compounds, different docking software have been developed, each with its own energy function and scoring function.^17−20^ For metal–organic compounds, most recent literature including docking does not explain how the parametrization of the metal atoms during simulations has been performed; alternatively, software may be used that is behind a paywall.^21^ To accelerate the field of metal-based medicinal compounds, it is of the utmost importance that open-source software be developed with which metal compounds can be docked in a cheap, easy, predictive, and reproducible manner.

In this article, we present MetalDock, an open-source Python program that can dock metal–organic compounds using the AutoDock docking engine.^22^ MetalDock utilizes a quantum software package for geometry optimization of the metal complex and charge determination prior to the docking itself. MetalDock is compatible with three widely used quantum software packages: ORCA,^23^ which is open-source; Gaussian;^24^ and Amsterdam Modeling Suite.^25^ Here, we used a Monte Carlo sampling scheme to obtain the necessary parameters required for docking molecular compounds based on 12 different metal centers. We show that MetalDock accurately targets the correct residue type for all metal compounds and that it can consistently reproduce X-ray structures of metal–organic compounds bound to proteins, with a root-mean-square deviation (RMSD) < 1 Å relative to the published experimental geometry. To validate the applicability of MetalDock, we performed three different case studies. The first case demonstrates that this program can be efficiently introduced in docking schemes, highlighting MetalDock’s precision in identifying potential sites where a coordination or covalent interaction could potentially take place. The second case highlights that the program is able to predict accurately the binding of coordinatively saturated metal complexes to protein kinases developed by the Meggers group.^26^ Finally, we show that MetalDock generalizes rather well as it can accurately dock metal–organic compounds to a DNA strand, a type of biomolecule that was not included in the training data set for the optimization of the docking parameters.

## Methods

### Metal Type Definition

**Scheme 1:**
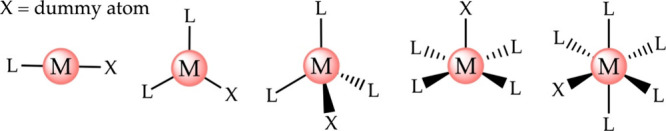
Schematic Figure to Exemplify Where the Dummy Atom Will Be Positioned if the Metal Center in the Metal-Organic Compound Is Di-, Tri-, Tetra-, Penta-, or Hexa-Coordinated

In AutoDock, an atom type can be created by defining several parameters that can essentially be separated into two classes: the intrinsic properties of the atom and the parameters that change the interaction of the metal atom with the protein, biomolecule, or DNA strand. In the former class, the van der Waals radii, the atomic solvation volume, and the van der Waals potential depth between two metal atoms needed to be specified. Although the former two could be retrieved from the literature for all metals,^27^ the latter is, unfortunately, hard to find. Metal–organic compounds typically contain a single metal center or multiple metal centers that are separated by a distance greater than the range of short-range van der Waals interactions. It was therefore decided that the van der Waals well depth between two metal atoms can be kept constant at the same low value as that of iron, i.e., 0.010 kcal/mol. A final intrinsic property parameter that needed to be specified was the hydrogen bonding capability of the new atom. Inspired by the paper of Sciortino et al.,^21^ we decided to set each metal atom to be a hydrogen bond donor, since hydrogen bonds and coordination bonds are both directional and polar. To activate this property, a dummy atom needed to be added to the metal–organic compound. MetalDock adds this dummy atom at the position of the vacant site in the coordination sphere, as exemplified in Scheme 1. The dummy atom has no charge nor any other physical property; it is solely added to activate the hydrogen bonding capabilities of the metal atom in AutoDock.

The short-range (repulsion plus attraction) van der Waals interaction of the metal atom with the protein, biomolecule, or DNA strand is usually described by several Lennard-Jones (LJ) potentials; see eqs 1 and 2, each containing two parameters. The protein can consist of seven different atom types: an aliphatic carbon (C), an aromatic carbon (A), a hydrogen that donates a hydrogen bond (HD), a nitrogen that accepts a hydrogen bond (NA), a nitrogen that does not accept a hydrogen bond (N), an oxygen that accepts a hydrogen bond (OA), and a sulfur that accepts a hydrogen bond (SA). Each interaction between the metal and atom types can be tuned by changing the well depth (ε in kcal/mol) and the minimum distance (*R*_min_ in Å) of the LJ potential. As we decided to describe the interaction of the metal and the protein to be like a hydrogen bond, we only needed to find the parameters for the metal center interacting with the OA, NA, and SA atom types. To describe the interaction of the metal with OA, NA, and SA as a hydrogen bond, the LJ potential was set to a 12–10 instead of a 12–6 potential. Under physiological conditions, cysteines are usually protonated. However, deprotonation of the sulfur atom can occur with the assistance of the metal atom, which leads to a coordination bond.^28^ To accurately approximate this coordination interaction of metal atoms with cysteines or other residues that can be deprotonated in such a metal-assisted manner, we added the HD metal atom interaction and used a 12–6 potential. We then minimized the number of parameters to be optimized by setting the *R*_min_ values of HD, NA, OA, and SA to 1.00, 2.20, 2.25, and 2.30, respectively, which we based on an analysis of the distances in each data set for each specific metal that we wanted to optimize and the size of each atom.
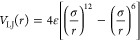
1
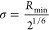
2

### Docking Protocol

**Scheme 2:**
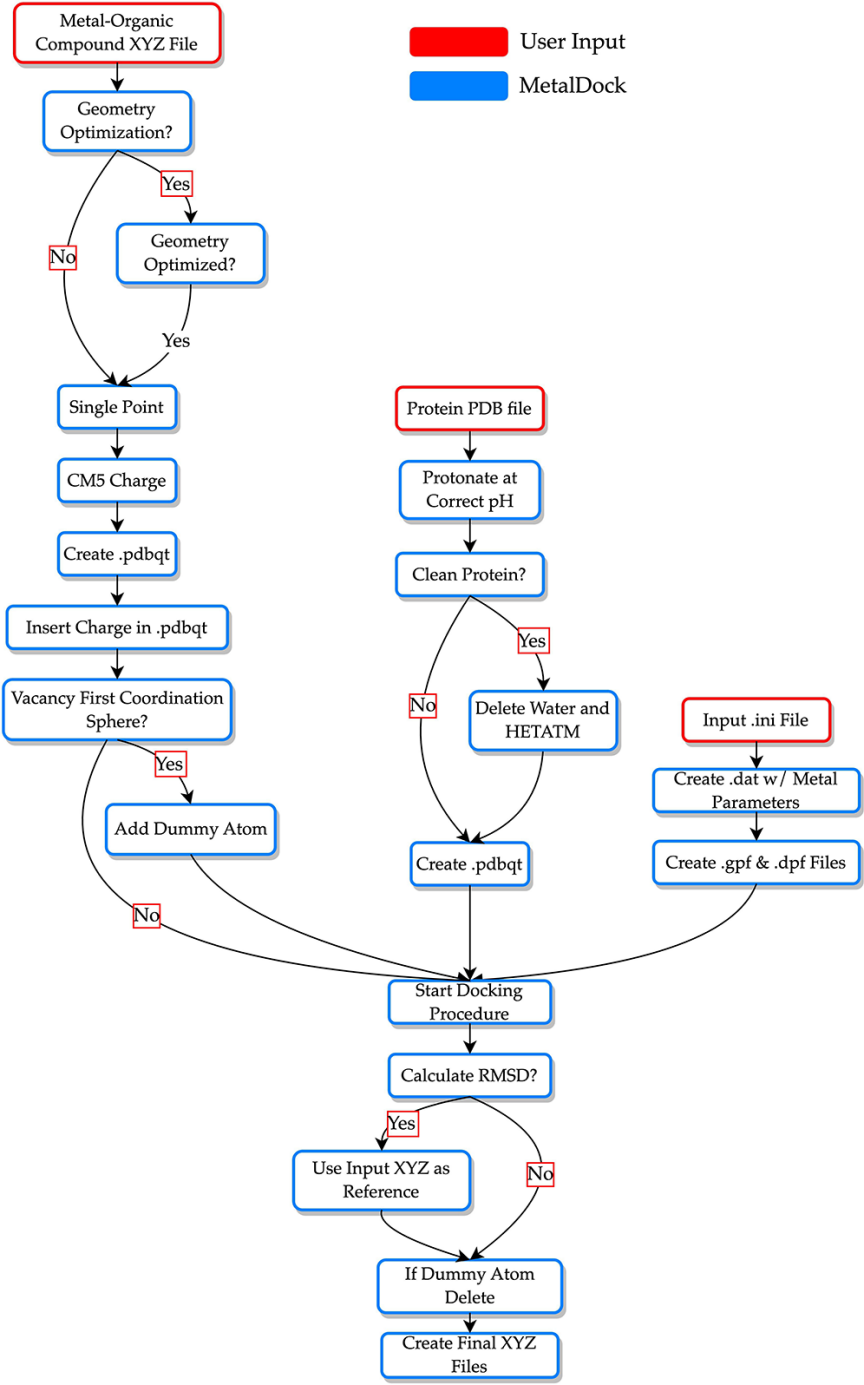
Workflow for the Docking Protocol of the MetalDock Program

The program workflow has been developed in such a way that the user needs to supply only an XYZ file of the metal–organic compound, a PDB file of the protein or nucleic acid strand, and an input file in which several parameters need to be specified, such as the charge and the multiplicity of the metal complex. The program will then operate following Scheme 2. It can be decided whether the geometry of the metal complex needs to be optimized first. This optimization as well as the subsequent single point calculation will be performed by one of the three different quantum software engines (ORCA, Gaussian, or ADF). Partial atomic charges will then be extracted from the single point calculation using the Charge Model 5 (CM5),^29^ after which these charges will be inserted into a pdbqt file. A dummy atom is then added to the molecule at the position where there is a vacant site in the first coordination sphere of the metal. If there is no vacant site or direct interaction between the metal and the protein, then the default parameter of adding the dummy atom needs to be set to false. Prior to the start of the simulation, the user can specify the pH of their experiment, and the protein will be protonated accordingly via the pdb2pqr software.^30^ The program can then delete all water and nonresidues if specified. However, if a cofactor plays a role in the binding of the docked metal–organic complex, this feature must be set to false to prevent the deletion of the cofactor. The protein will also be converted to a pdbqt file. The box size, as well as the docking center and other docking parameters, need to be specified in the input file. All files that are necessary for docking are then created, and the docking procedure will start. Finally, if the metal–organic complex inside the protein has already been determined experimentally, the user can request to calculate the root-mean-square deviation (RMSD)^31^ between the docked poses and the experimentally determined coordinates of the metal–organic atoms.

### Data Set

A data set was created for each of the 12 metal atom types: Cr, Co, Cu, Mo, Ni, Os, Pd, Pt, Re, Rh, Ru, and V. Our original goal was to incorporate as many transition metals as possible, but only these 12 atom types had more than two X-ray structures in the protein data bank (PDB) featuring metal–organic compounds interacting with proteins. Notably, other metals, such as Au, had several crystal structures in the PDB, but these structures did not have any ancillary small-molecule ligand bound to the metal. In such cases, the metal fragment was limited to a single atom (the metal ion) and hence it did not have any torsional degree of freedom, rendering it unsuitable for docking. In future research, we aspire to extend the scope of this program to other atom types, ultimately encompassing all transition metals, but more crystal structures are needed in the PDB. Each data set was used to obtain the well depth of the LJ interaction of the four unspecified interactions between the metal and the involved protein’s atom types (NA, OA, SA, and HD). Each data set consisted of all X-ray structures found in the PDB that describe a metal–organic compound interacting with a protein. Metal–organic compounds that had more than 10 torsional degrees of freedom were left out, as AutoDock cannot reproduce consistent results for such compounds. We protonated each metal–organic compound according to the correct protonation state at the pH used during crystallization. The structure of the metal–organic compound was extracted from the PDB and these coordinates were later used in a reference file for comparison of the docked poses. The number of compounds included in the data set for different metals can be seen in Table 1. No DNA structures were included. Each metal–organic compound underwent conversion into a bit string, enabling a comparison between individual inputs using the Tanimoto similarity test.^32^ This test is defined as the ratio of the intersection to the union of bits between two compounds, as outlined in eq 3:
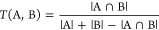
3Here, |A∩B| represents the overlap of bits between compounds A and B. |A| and |B| are the total number of bits in compounds A and B, respectively. All corresponding proteins were cross-referenced using a template modeling (TM) score.^33^ The TM score addresses two key issues found in conventional metrics such as RMSD. Firstly, it prioritizes smaller distance errors over larger ones, enhancing sensitivity to global fold similarity rather than local structure variations. Secondly, it incorporates a length-dependent scale to normalize distance errors, ensuring length independence of the TM score for random structure pairs. For both tests, the value ranges from 0 to 1, where 1 indicates an identical pair.

**Table 1:** Different Metal Atoms for Which the LJ Parameters Were Found and the Number of Data Points

atom	*N*	atom	*N*	atom	*N*
Cr	3	Ni	2	Re	10
Co	11	Os	4	Rh	3
Cu	6	Pd	7	Ru	11
Mo	11	Pt	10	V	4

### Determination of Atomic Charges and Lennard-Jones Parameters

Quantum mechanical calculations were performed to determine the charges on the atoms of the metal–organic compound. The quantum calculations were performed with the AMS 2021 package.^25^ CM5 charges were used that were extracted by taking a single point of each metal–organic compound at the TZP^34,35^/B3LYP^36^/COSMO^37^ level, including the Grimme’s dispersion corrections D3 with BJ damping.^38^ Relativistic effects were scalarly corrected for by ZORA.^39^ We assumed a low spin configuration for all metal complexes. As the well depth (ε) parameter of the LJ interaction of the metal and the atom types of the protein was not available in the literature, we used a Monte Carlo sampling scheme to find the optimal values. The optimization scheme started with an ε of 2 kcal/mol for each interaction (ε NA, ε OA, ε SA, and ε HD). During a cycle, an ε value was sampled from a continuous equiprobable probability distribution that ranged from 0 to 7 kcal/mol. The sample replaced the current ε value, and all metal–organic compounds in the training set were docked with a box size of 20 × 20 × 20 Å to their proteins with the new parameters. At the start of the docking procedure, the metal–organic compound was randomly rotated and translated within the specified box to prevent any bias to its initial position. Subsequently, the genetic algorithm implemented in AutoDock was used to search for the conformation with the lowest potential energy. This was repeated 10 times to finally obtain 10 different poses. During docking, the protein atoms were kept fixed, and eventually the all-atom RMSD between the X-ray structure coordinates of the metal–organic compound and the 10 docked poses was calculated. Finally, a total RMSD was obtained following eq 4:
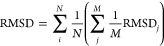
4where *N* is the number of protein metal–organic complex pairs and *M* is the number of poses calculated for each protein metal–organic complex pair. The LJ parameter was then accepted according to the following criteria:
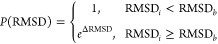
5
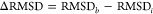
6Here, RMSD_*i*_ is the RMSD obtained from iteration *i* and RMSD_*b*_ is the RMSD of the previously accepted parameter set. Each parameter was separately sampled 250 times. The optimized LJ parameters are reported in Table S1.

## Results

### Unique Data Points

**Figure 1:**
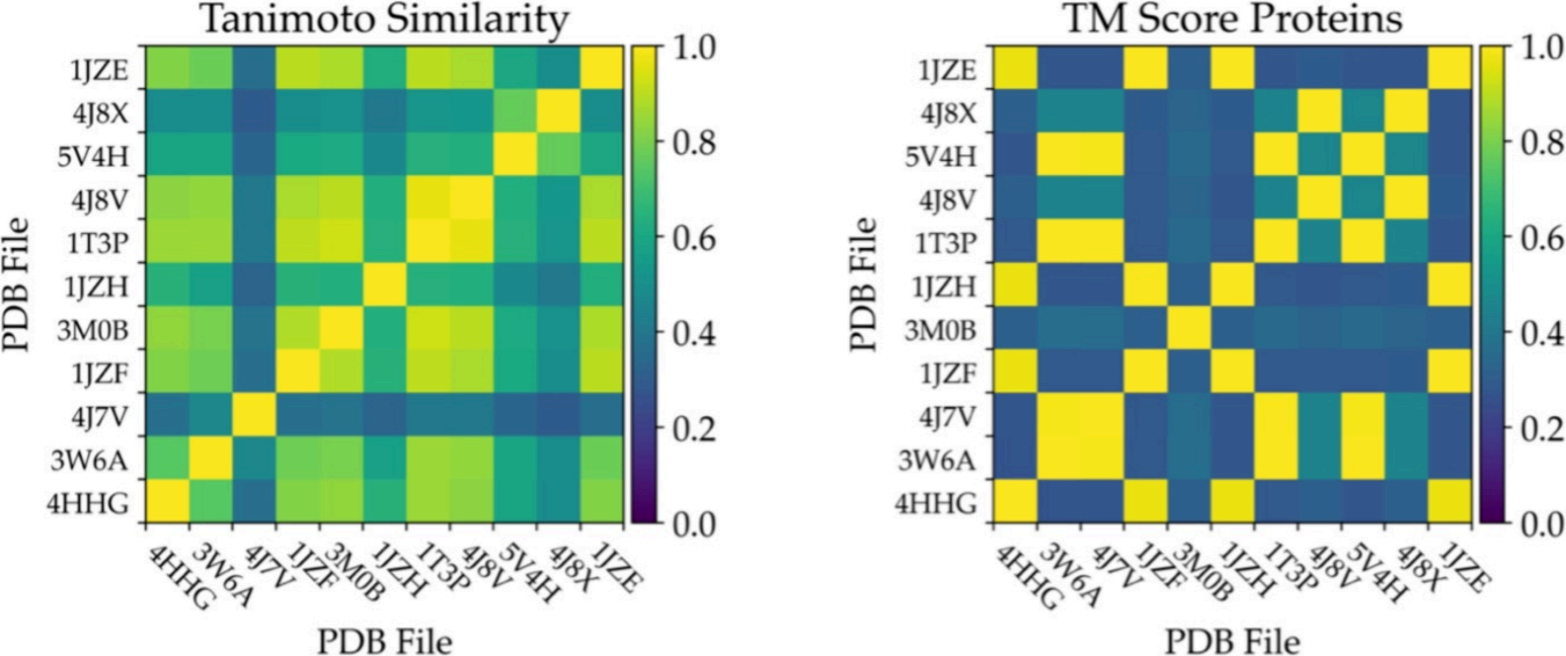
Tanimoto similarity and the TM scores between the different PDB files used for the docking of metal–organic compounds that contain ruthenium.

For each metal atom type, to predict the generalizability of our parameters we first calculated the Tanimoto similarity between all metal–organic compounds in the data set, which included the metal atom type of interest and the template modeling (TM) score between all corresponding proteins.^33^ The protein metal–organic complex pairs that in total differed the most from the rest of the data set were chosen as a test sample. In Figure 1, the calculated values for all PDB files of the metal–organic compounds that contained ruthenium are plotted. From these calculations, it became clear that 4J7V differed structurally the most; it was hence chosen as test sample and was thus not used as a data point in the optimization of the Lennard-Jones parameters. The calculations for the other metal atom types can be found in Figures S1–S11.

### Parameter Optimization

The optimization by Monte Carlo sampling led to LJ parameters that could reproduce the X-ray structure of the metal complexes accurately. In Table 2, the average RMSD of the best parameter set can be seen, as well as the average resolution of the X-ray structures. This latter property is the inherent error in the experimental data. The obtained parameters for the metals Cr, Co, Mo, Ni, Pd, Re, Rh, Ru, and V could reproduce the conformation of the metal complex within the experimental uncertainty, as their average RMSD was lower than the average RMSD of the resolution. A lower RMSD in comparison to the resolution would not indicate that MetalDock could more accurately predict the conformation of a metal complex.

**Table 2:** Average RMSD of the Best Parameter Set Found by Monte Carlo Sampling

atom	RMSD (Å)^a^	resolution (Å)^a^	atom	RMSD (Å)^a^	resolution (Å)^a^	atom	RMSD (Å)^a^	resolution (Å)^a^
Cr	0.750	1.643	Ni	1.208	2.500	Re	1.620	1.786
Co	1.655	1.757	Os	2.228	1.720	Rh	0.775	1.950
Cu	2.501	2.402	Pd	1.905	1.822	Ru	1.222	1.854
Mo	1.314	2.094	Pt	2.645	1.944	V	0.716	1.275

a The average of all the protein metal–organic complex pairs that were used during the parameter optimization procedure.

After optimization, only for Cu, Os, and Pt did MetalDock exhibit a higher RMSD than experimental resolution. On the other hand, the difference between these two values was rather small for all three metals: 0.099, 0.508, and 0.701 Å, respectively. A RMSD lower than 1 Å with respect to the crystallographic structure is considered excellent, which is what we observed for all metals.^40^

Upon examining the optimized parameters (Table S1) more closely, we noticed that certain metal atom types, which had a relatively limited number of experimental data points, displayed a degree of insensitivity in their parameters. In the case of Ni, for instance, we observed that each metal–organic complex interacted with a histidine. Although other atom types were present during docking, the well depth of the OA and SA parameters had a negligible impact on the RMSD. The metal–organic compounds that contained a Mo atom predominantly exhibited interactions with cysteines or tyrosines. The accurate description of this interaction heavily relied on the 12–6 covalent term between the HD atom type and the metal (5.62 kcal/mol), as opposed to the SA interaction (0.17 kcal/mol). A similar result was observed for Os, where one metal–organic complex interacted with an asparagine residue. Once again, the HD term dominated to describe the correct interaction between the residue and the metal–organic complex. Under the pH of each experiment, the residues (Cys and Asn) are predicted to be protonated by propka.^41^ As explained earlier, for exactly that reason, we added the HD parameter to accommodate metal-assisted deprotonation in the docking scheme. This feature was prevalent across almost all metal atom types, where the low well depth of a specific atom type was occasionally present due to the presence of hydrogen atoms on residues that experimentally would be removed in a metal-assisted manner. Our results exemplify the necessity of this parameter to accurately describe the orientation of the poses.

### Generalization of MetalDock

**Figure 2:**
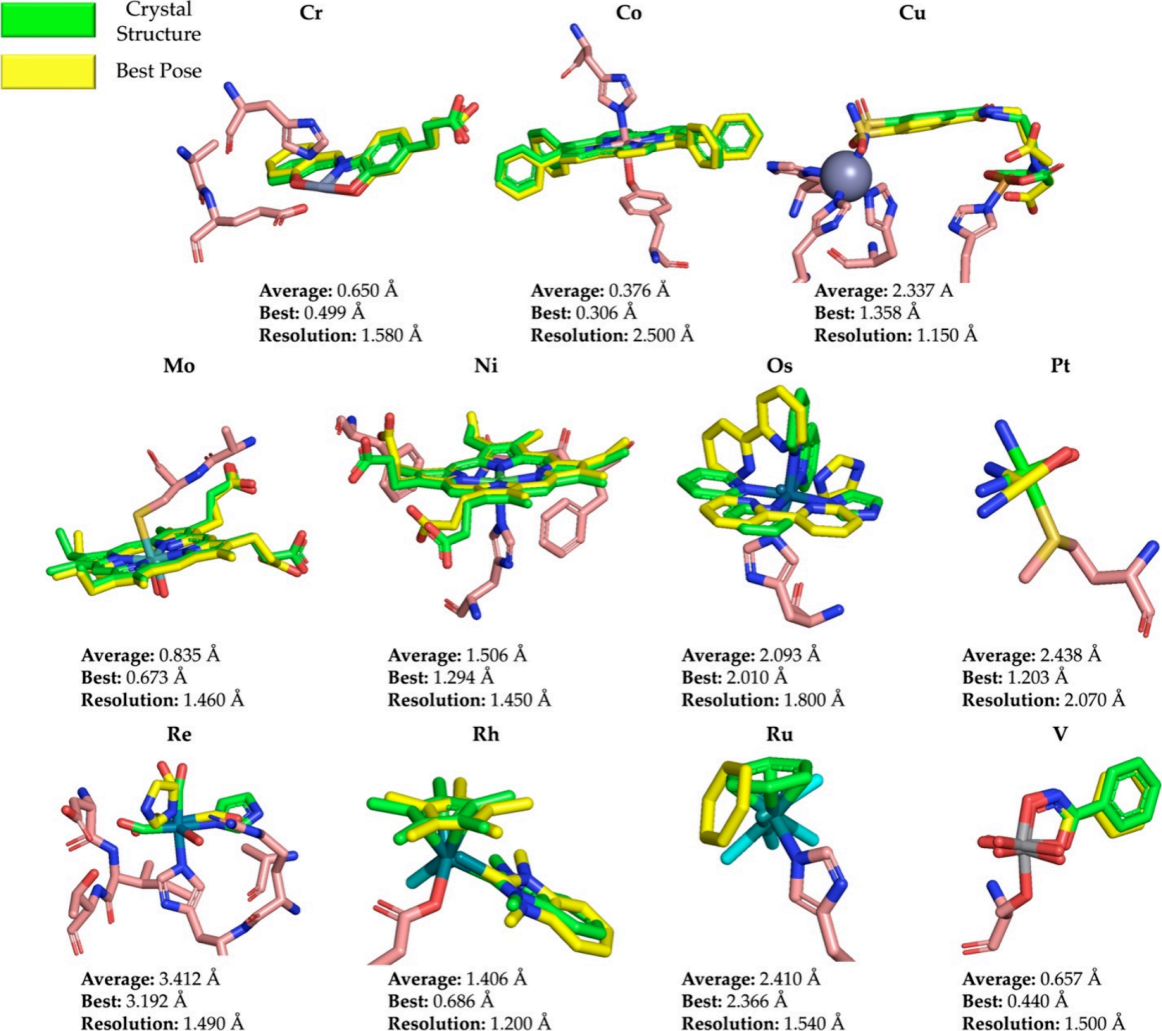
Superposition of the X-ray structure and the best pose obtained by MetalDock for the different metal complexes used as a validation run. The green structure is the metal complex in the X-ray structure, the yellow structure is the MetalDock pose that had the best RMSD, and the pink residues represent the protein residues that interacted more strongly with the metal–organic compound in the best pose.

**Figure 3:**
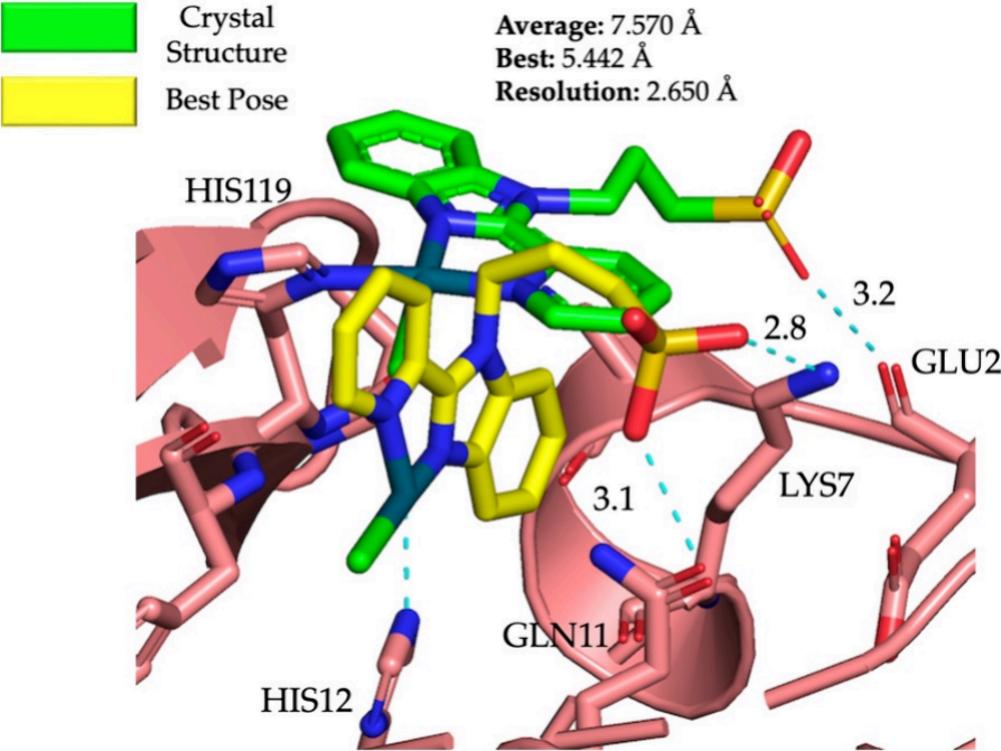
X-ray structure and the best pose of the Pd metal complexes that were used as a validation run. The green structure is the metal complex in the X-ray, the yellow structure is the pose that was obtained with the best RMSD, and the pink residues represent the protein residues that interacted more strongly with the metal–organic compound.

To verify the generalizability of MetalDock, we docked the most different protein metal–organic complex pair, based on our similarity scores, that was not included in the data set for the parameter optimization. We observed that almost all metal complexes would be docked to the same residue as that of the crystal structure, although there were some significant differences between different metals. In Figure 2, the best pose of the MetalDock run, the X-ray structure of the metal complex and the nearby residues can be seen for each metal test set. In the context of these test protein metal–organic complex pairs, the parameters for the metals Cr, Co, Mo, and V generalized the best. For each of these metals, the average of the RMSD of the 10 docked poses was lower than the resolution of the X-ray structure. The poses of the test set of metals Ni, Pt, and Rh resulted in the best pose that was lower than the resolution, although the average was slightly higher. With a difference of 0.056, 0.368, and 0.206 Å, respectively, MetalDock was still able to consistently reproduce a comparable conformation with respect to the X-ray structure. The docking results for the metals Cu, Os, and Ru showed a slightly higher RMSD but were still considered excellent as the total RMSD was lower than 1 Å when compared to the crystallographic structures. The differences between the resolution and the best pose were 0.208, 0.210, and 0.826 Å for Cu, Os, and Ru, respectively. The poses of the test set of Re had a relatively high RMSD, even though all poses were found to be attached to the correct residue, His15. The main reason for the high RMSD was a 180° rotation of the metal complex, resulting in the formation of a hydrogen bond between the pyridine ligand and the Asp87 residue instead of the CO ligand. The only atom type in which we saw that the metal complex interacted with a different residue than found in its crystal structure was the Pd atom type (Figure 3). However, the residue type remained the same as the Pd atom interacted with His19 instead of His119. In the X-ray structure, the sulfoxide tail interacts with a hydrogen bond with the carboxylate of Glu2.^42^ MetalDock predicts that it can form a similar hydrogen bond with Gln11, and in addition, it finds a second possible hydrogen bond with Lys7. The extra hydrogen bond provides an explanation of why conformations that lead to these two interactions are more favorable in the potential energy landscape as compared to the X-ray structure.

### Case Study 1: Enantioselective Rhenium Inhibitor

**Figure 4:**
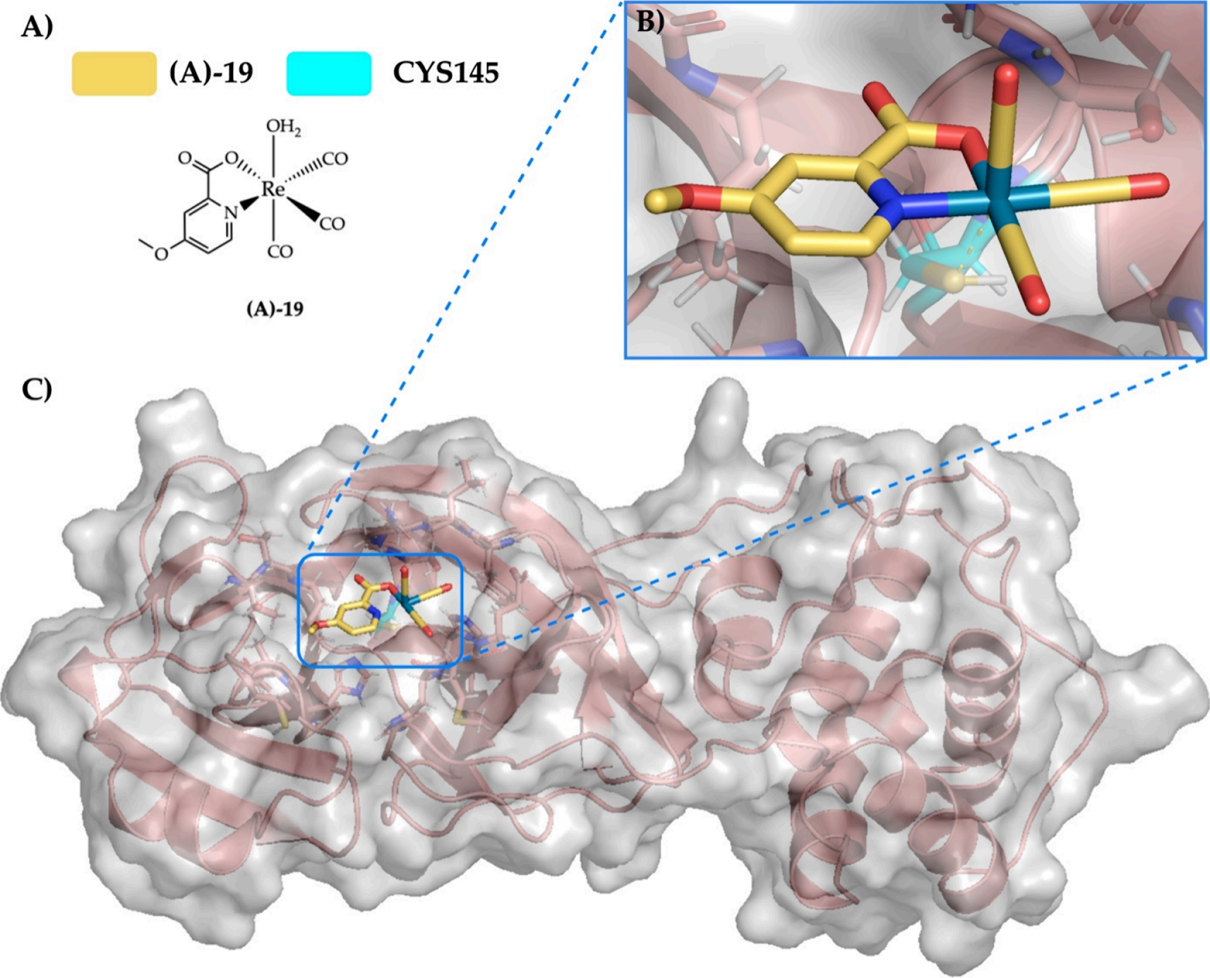
(A) The active enantiomer of the rhenium complex that was tested for inhibition of the 3CL^pro^ protein by Karges et al.^8^ (B) Upon docking with MetalDock, the **(A)-19** enantiomer was found to interact with the Cys145 residue in a form of covalent inhibition. (C) PDB code: 6Y2F. Color code: Re (turquoise), O (red), N (blue), and C (yellow). The gray area is the van der Waals surface of the atoms of the protein.

To test the predictive power of MetalDock, we performed a case study on a recently discovered enantioselective rhenium inhibitor.^8^ The main target of this inhibitor was the 3-chymotrypsin-like protease (3CL^pro^), which is considered to be one of the main targets of the SARS-CoV-2 virus. A series of covalent rhenium inhibitors were synthesized by Karges et al.,^8^ who observed that the **(C)-19** enantiomer had a high IC_50_ (57 ± 9 μM) and thus a low activity, whereas the **(A)-19** enantiomer covalently bound to Cys145, which resulted in a remarkably low IC_50_ (1.8 ± 0.3 μM). This primarily experimental study also employed a covalent docking protocol that did not consider the metal explicitly. In order to assess MetalDock’s predictions, we conducted docking experiments involving the **(A)-19** enantiomer with three different box sizes (40 × 40 × 40 Å, 60 × 60 × 60 Å, and 100 × 100 × 100 Å), all centered on the Cys145 residue. Prior to docking, we optimized the metal–organic compounds as no crystal structure existed that included this compound and only PDB files of solely protein were available. We used the same level of theory as the protein metal–organic complex pairs in the data set (TZP^34,35^/B3LYP^36^/COSMO^37^/D3-BJ damping^38^/ZORA^39^). In solution, the water ligand will dissociate from rhenium, and a vacancy in the first coordination sphere will facilitate a direct interaction between the metal and the protein cysteine residue. The result of the docking of the 40-sided box with MetalDock can be seen in Figure 4: as in the Karges paper, all 10 docked poses of the **(A)-19** enantiomer were found to interact with the sulfur of the Cys145 residue, which would lead to a coordination bond if electrons were considered explicitly (for example in a QM/MM model). MetalDock implicitly takes into consideration these coordination sites, eliminating the need for an extended covalent docking protocol, which does not necessarily improve the accuracy of the prediction.^43^ Expanding the box dimensions to 60 and 100 Å on each side during the docking process resulted in five and one interactions out of 10 poses with the Cys145 residue, respectively. This result particularly emphasizes the ability of MetalDock to accurately identify possible sites where a coordination interaction with a protein residue can take place.

### Case Study 2: No Vacancy in the First Coordination Sphere

**Figure 5:**
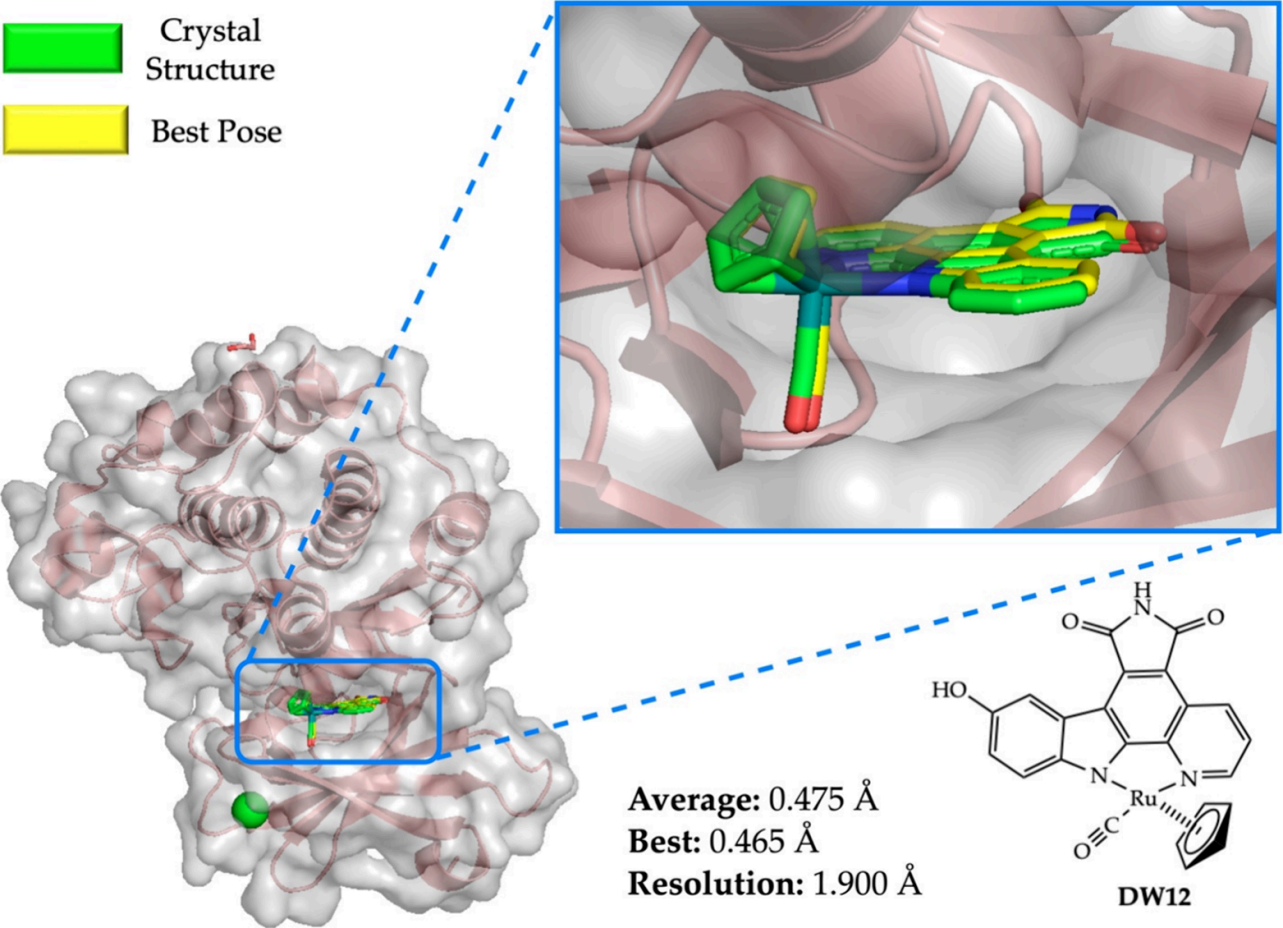
Docking with MetalDock of the **DW12** protein kinase inhibitor with its PIM1 target. The average RMSD was calculated from the 10 best poses. PDB code: 2BZH. Color code: Ru (turquoise), N (blue), O (red), C (green for X-ray, yellow for best pose). The gray area is the van der Waals surface of the atoms of the protein.

MetalDock also incorporates the option to dock metal–organic compounds where the metal center is thought of as a hypervalent carbon atom. These types of compounds do not have any vacancy in their first coordination sphere so that the metal does not directly interact with the protein via the formation of a coordination bond. To verify if our program could also reproduce the correct conformations for such compounds, we investigated the interaction between Meggers’ metal-based inhibitor **DW12** and its target, the protein kinase PIM1.^12^ The box size was set to 20 × 20 × 20 Å, and in the input file of MetalDock, the vacant site option was set to false to ensure that no dummy atom was added to the compound. The single point for the structure was taken at the same level as the data set (TZP^34,35^/B3LYP^36^/COSMO^37^/D3-BJ damping^38^/ZORA^39^). The final best pose of the docking procedure and the X-ray crystal structure of the **DW12**–PIM1 adduct are superimposed in Figure 5. We obtained an excellent average RMSD in this case (0.475 Å for the average best 10 poses and 0.465 Å for the best pose) that was lower than the resolution of the crystal structure (1.9 Å). This result indicated that MetalDock could perfectly reproduce the experimental binding geometry of this compound to the protein.

### Case Study 3: DNA Strand

**Figure 6:**
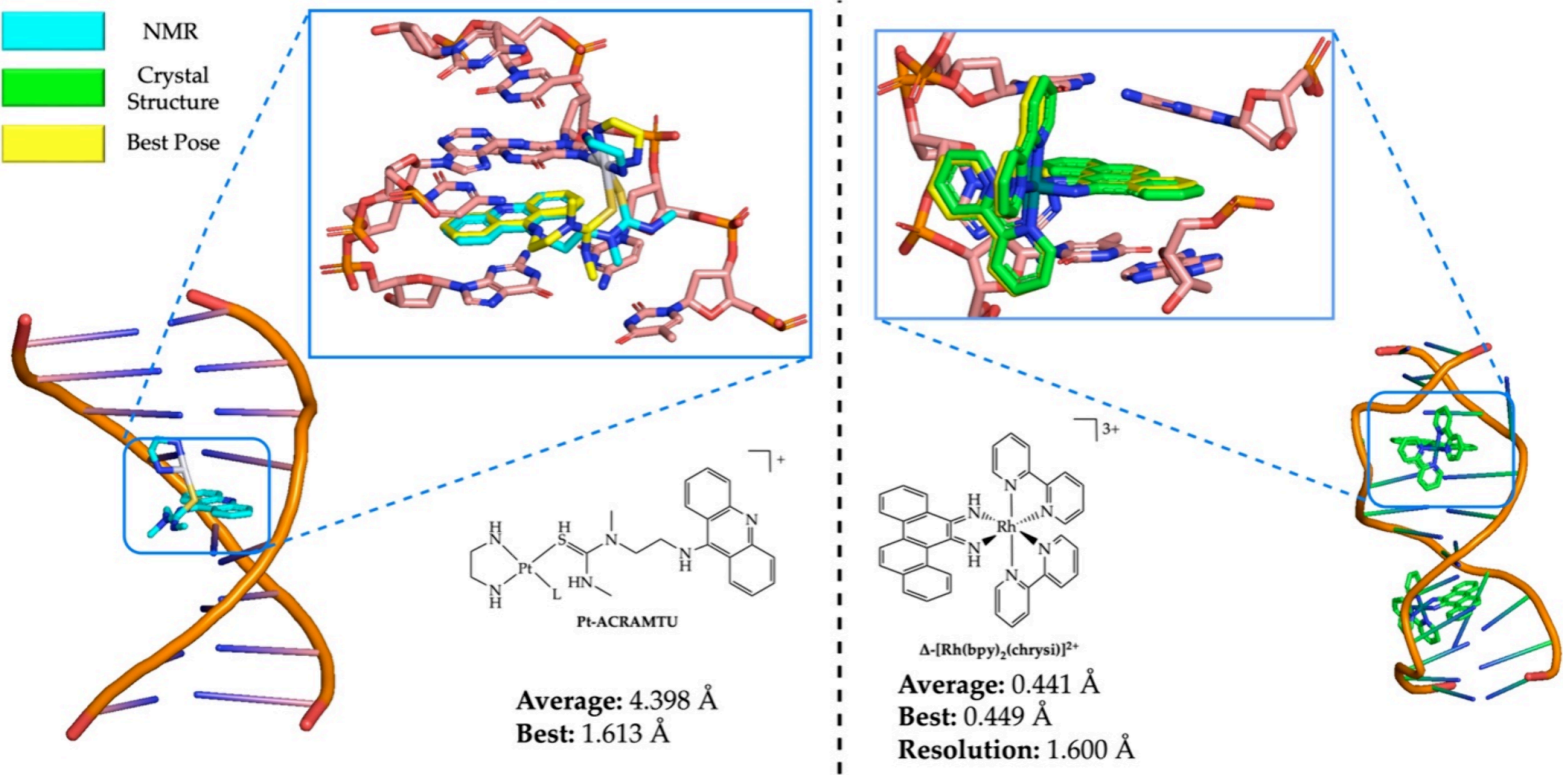
Docking of metal complexes with DNA strands using MetalDock. (left) Interaction between the [Pt(en)ACRAMTU)]^3+^ compound and the octamer strand 5′-CCTCG*TCC-3′/3′-GGAGCAGG-5′, where the asterisk indicates the platinum fragment. L is H_2_O or N7 of the G* residue. The full view of the DNA–metal complex shows the NMR structure. PDB code: 1XRW. Color code: Pt (silver), N (blue), S (gold), and C (cyan for NMR, yellow for best pose). (right) The interaction between [Rh(bpy)_2_(chrysi)]^3+^ and the oligonucleotide duplex 5′-CGG*A*AATT*A*CCG-3′ containing two adenosine–adenosine mismatches (italics). PDB code: 3GSK (X-ray crystal structure). The average RMSD was calculated from the 10 poses. The full view of the DNA–metal complex shows the X-ray structure. PDB code: 3GSK. Color code: Rh (turquoise), N (blue), and C (green for X-ray, yellow for best pose).

As there were no DNA structures included in the training set, we wanted to verify if MetalDock would also be able to accurately dock metal–organic compounds to DNA. Luckily, as we did not use any DNA strands or even biomolecular structures obtained via NMR, there was still some reference material left in the PDB. We decided to dock two metal–organic compounds, one where one of the nucleotides binds directly to the metal atom and one metal–organic compound where there is no vacancy in the first coordination sphere of the metal. For the former case, we investigated the coordination of [Pt(en)ACRAMTU)]^3+^ (en = 1,2-ethylenediamine, ACRAMTU = 1-[2-(acridin-9-ylamino)ethyl]-1,3-dimethylthiourea) to a DNA octamer strand reported by Baruah et al.^44^ The PDB structure was obtained via NMR (only one conformer submitted) and did not contain any Mg^2+^ or Na^+^ cations, which are known to stabilize the negatively charged phosphate groups of the backbone in aqueous solution. Consequently, if we would include the phosphate groups in the box size, the positively charged metal would always favor interaction with the negatively charged oxygens. To circumvent this problem, we shifted the 20 × 20 × 20 Å box so as not to include the phosphate backbone. Single points were taken at the same level of the data set (TZP^34,35^/B3LYP^36^/COSMO^37^/D3-BJ damping^38^/ZORA^39^) and a total of 10 best poses were generated by MetalDock. Among these, we obtained a remarkably accurate pose wherein Pt exhibited a coordination interaction with N7 of the G residue in the 5′ to 3′ strand. This coordination was highly similar to the NMR structure, with a deviation of merely 1.613 Å from the NMR structure, as illustrated in Figure 6 (left). In the second case, we docked Barton’s [Rh(bpy)_2_(chrysi)]^3+^ (chrysi = 5,6-chrysenequinone diimine) to an adenosine–adenosine DNA mismatch strand that was obtained via X-ray crystallography.^45^ As there was no vacancy in the first coordination sphere of [Rh(bpy)_2_(chrysi)]^3+^, the phosphate backbone could be included within the box. Once again, we used a 20 × 20 × 20 Å box size. We achieved an average RMSD between the 10 poses and a crystal structure of 0.441 Å, surpassing the resolution of the crystal structure itself (1.6 Å, see Figure 6 (right)).

## Conclusion

Our study highlighted the effectiveness of MetalDock in accurately docking metal–organic compounds to biomolecules such as proteins and DNA oligomers through the utilization of a Monte Carlo optimization scheme. While the comparisons to experimental results were excellent, there is still room for improvement in increasing the number of data points in each experimental data set used for the training. As new PDB entries on metal–organic compounds interacting with biomolecules are published, this problem will be addressed. To facilitate the evolution of our program, we have made the optimization and test procedure also available on GitHub (https://github.com/MatthijsHak/MetalDock), allowing future users to optimize the current parameters or expand the scheme to include new metal atom types. The versatility of this tool was exemplified through the three case studies presented, showcasing its ability to predict the binding of metallodrugs with high accuracy. Furthermore, the docking parameters optimized from X-ray crystal structures were found to generalize rather well, as the program successfully docked compounds to DNA strands based on NMR data, which were not present in the original data set used for parameter optimization. With this program, we hope to provide a useful tool to the bioinorganic community that will allow to quickly and accurately predict the interaction between metallodrugs with an open or closed coordination sphere and proteins or DNA strands.

## Supporting Information Available

It contains the final parameters for all different metals as well as the similarity test of the metal–complex pairs (PDF)
