# Supplementary material for: MetalDock: An Open Access Docking Tool for Easy and Reproducible Docking of Metal Complexes
Source: J Chem Inf Model. 2023 Dec 4;63(24):7816–25. doi: 10.1021/acs.jcim.3c01582 (PMC10751784; doi:10.1021/acs.jcim.3c01582)
Supplement: Supplementary file 1 — ci3c01582_si_001.pdf [file ci3c01582_si_001.pdf]

Supporting Information

MetalDock: an open access docking tool for easy and reproducible docking of metal complexes

Matthijs L. A. Hakkennes,[†] *Francesco Buda,* [*†] *Sylvestre Bonnet**[†]

[†] Leiden Institute of Chemistry, Leiden University, P.O. Box 9502, 2300 RA Leiden, The Netherlands

*Corresponding author: *bonnet@chem.leidenuniv.nl* , *buda@chem.leidenuniv.nl*

Keywords: bioinorganic, metallodrugs, proteins, drug design, Monte Carlo, docking

NO TABLE OF CONTENTS ENTRIES FOUND.Lennard Jones Parameters Atom Types

Table S1. The parameters obtained after the Monte Carlo sampling scheme for the different atom types.

Metal	ε NA (kcal/mol)	ε OA (kcal/mol)	ε SA (kcal/mol)	ε HD (kcal/mol)
Cr	6.371	1.998	0.144	3.625
Co	5.280	0.050	6.673	5.929
Cu	4.696	1.277	6.791	1.114
Mo	1.330	0.014	0.168	5.620
Ni	0.630	2.732	4.462	2.820
Os	5.958	0.135	4.102	6.589
Pd	4.688	0.845	5.574	3.159
Pt	6.532	2.020	6.332	1.844
Re	6.738	0.645	3.309	4.502
Rh	5.559	2.056	0.573	5.471
Ru	6.936	2.796	4.295	6.357
V	4.696	6.825	5.658	3.984

Metal Complex and Protein Similarity Test

Chromium

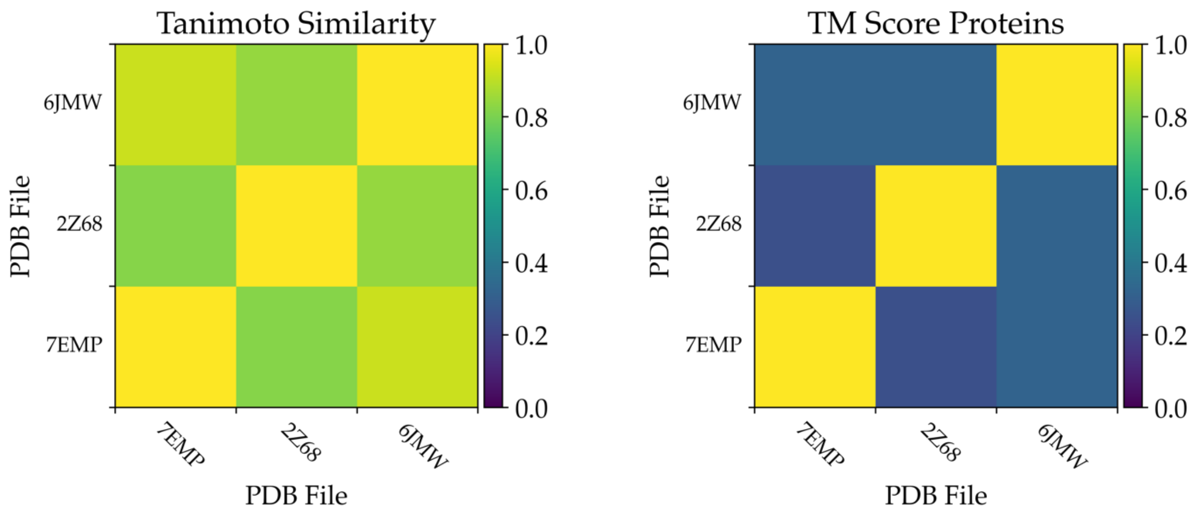

Figure S1. The Tanimoto similarity and the TM-score between the different PDB files used for the docking of organometallic compounds that contain chromium.

Cobalt

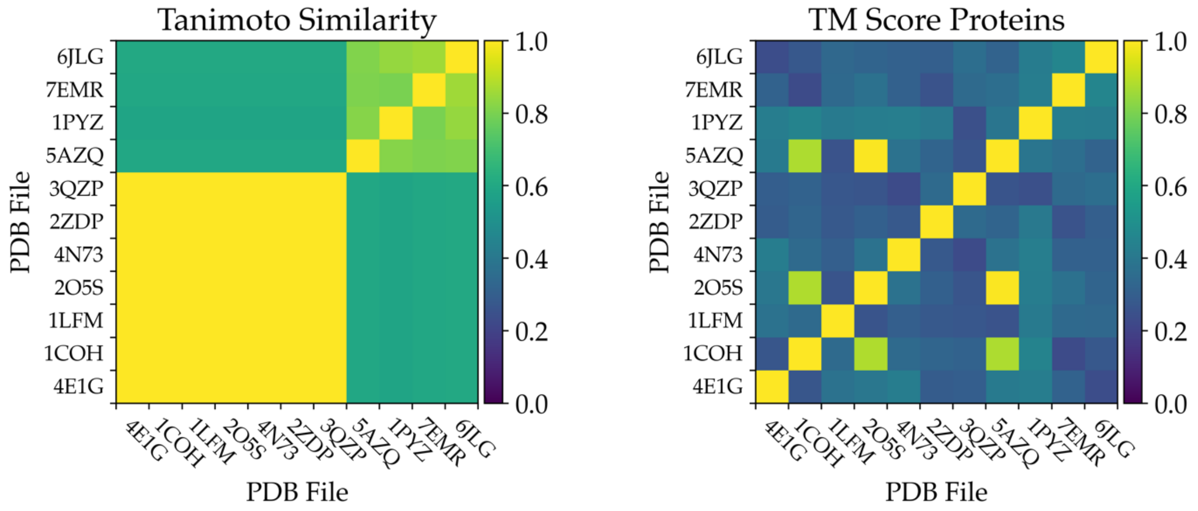

Figure S2. The Tanimoto similarity and the TM-score between the different PDB files used for the docking of organometallic compounds that contain cobalt.

Copper

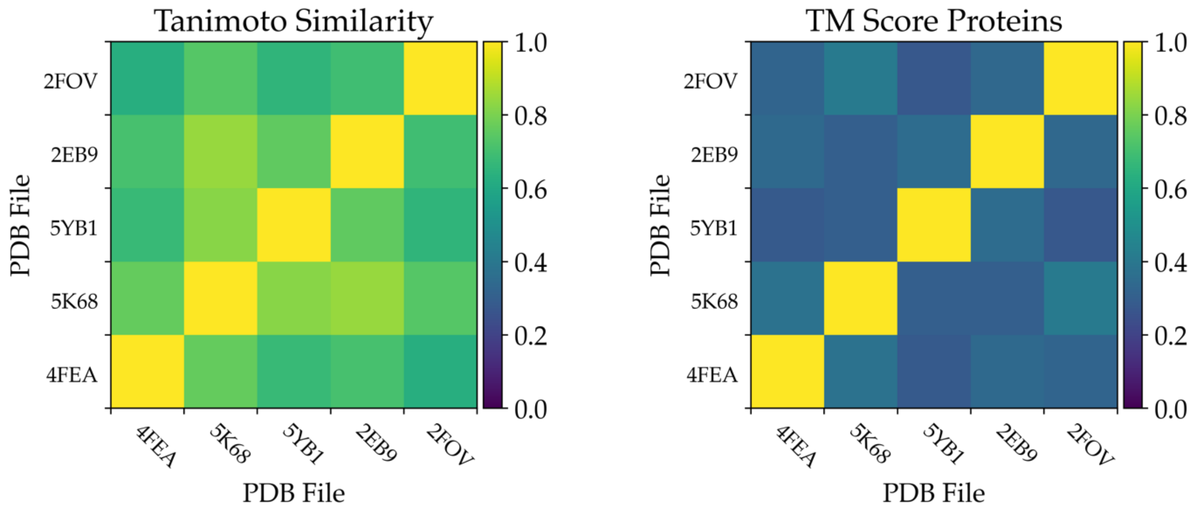

Figure S3. The Tanimoto similarity and the TM-score between the different PDB files used for the docking of organometallic compounds that contain chromium.

Molybdenum

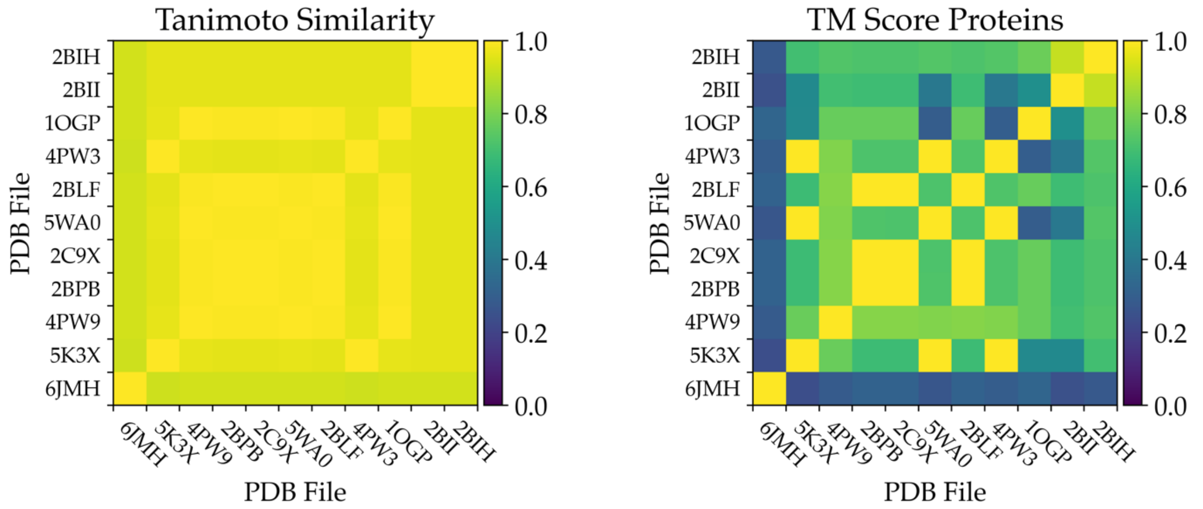

Figure S4. The Tanimoto similarity and the TM-score between the different PDB files used for the docking of organometallic compounds that contain molybdenum.

Nickel

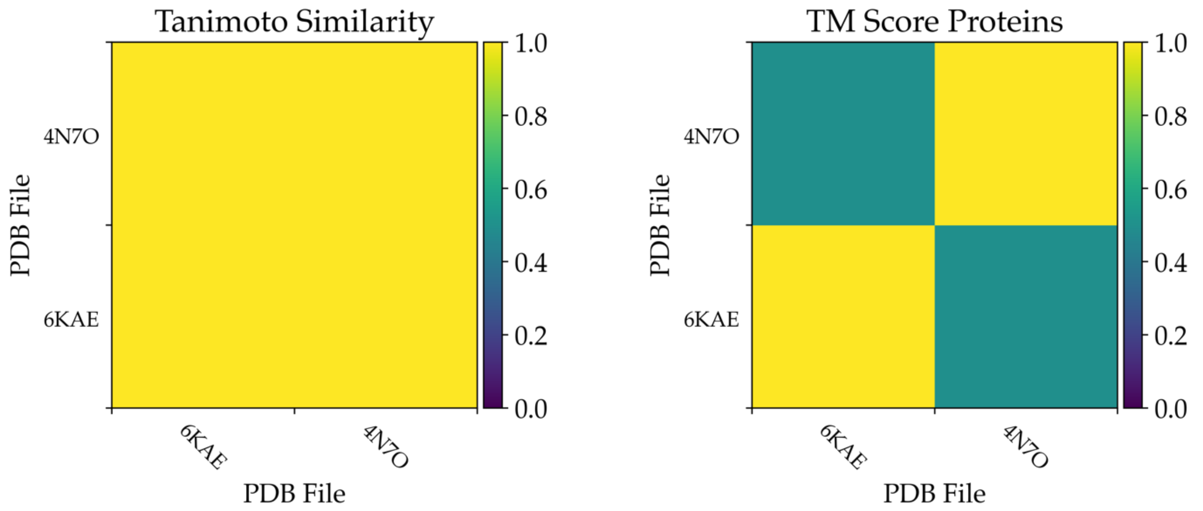

Figure S5. The Tanimoto similarity and the TM-score between the different PDB files used for the docking of organometallic compounds that contain nickel.

Osmium

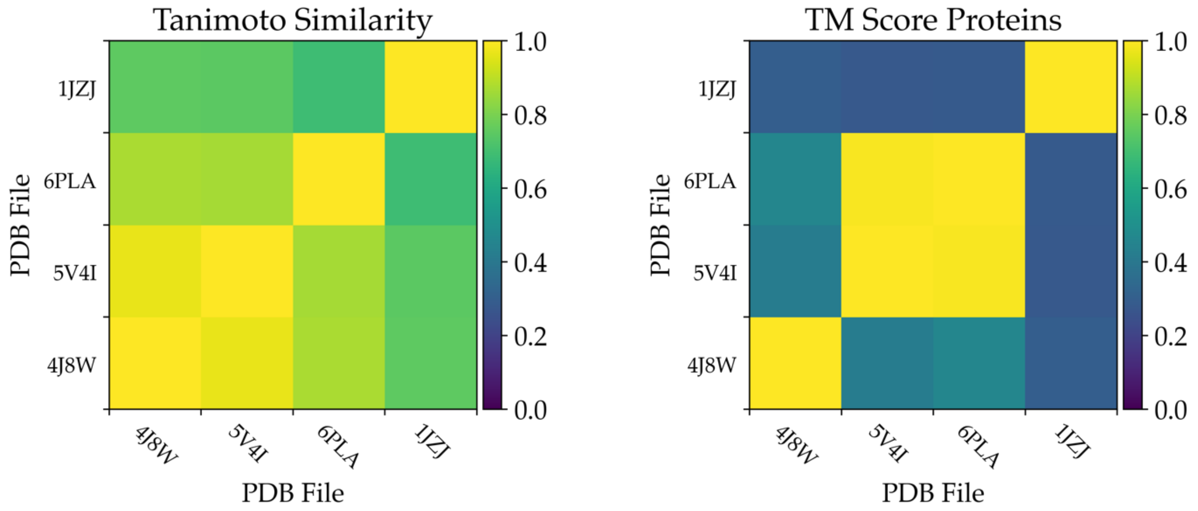

Figure S6. The Tanimoto similarity and the TM-score between the different PDB files used for the docking of organometallic compounds that contain osmium.

Palladium

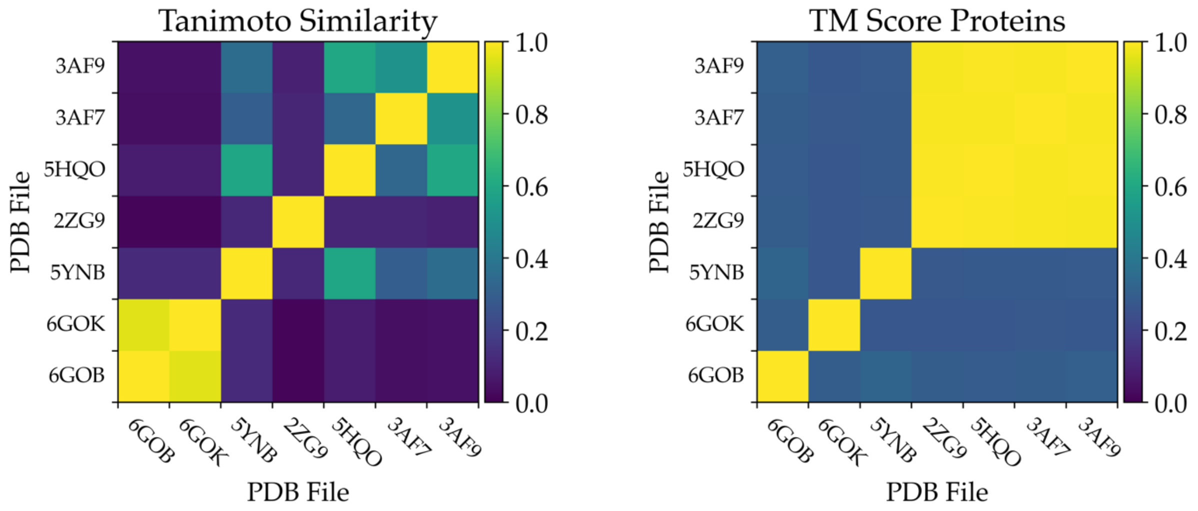

Figure S7. The Tanimoto similarity and the TM-score between the different PDB files used for the docking of organometallic compounds that contain palladium.

Platinum

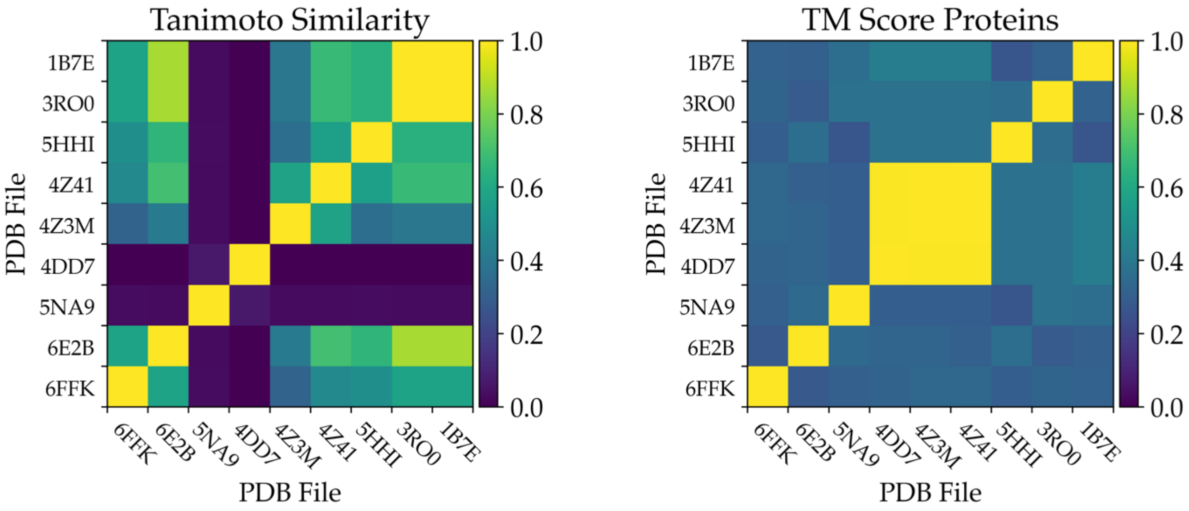

Figure S8. The Tanimoto similarity and the TM-score between the different PDB files used for the docking of organometallic compounds that contain platinum.

Rhenium

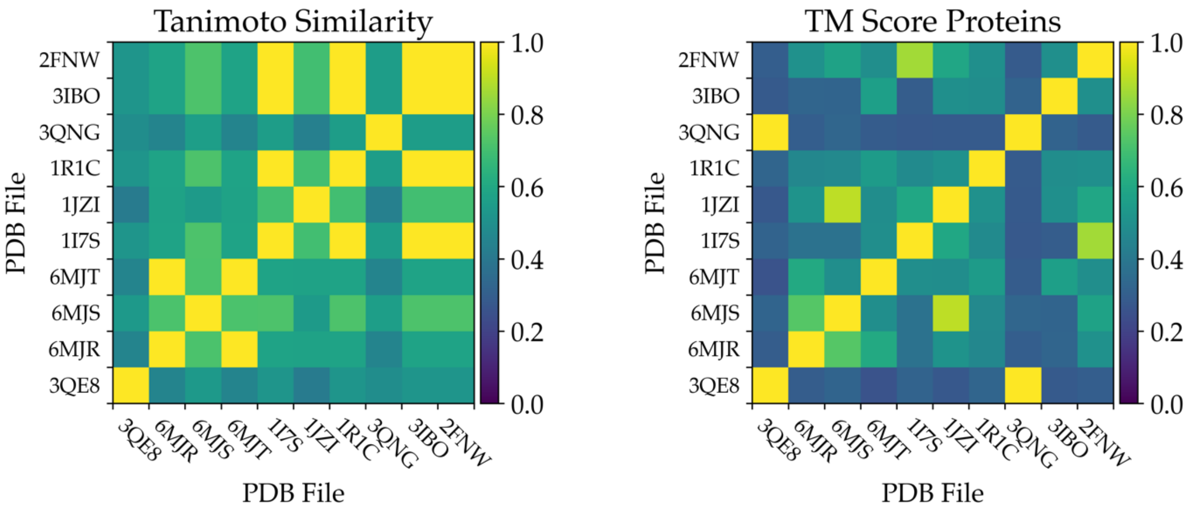

Figure S9. The Tanimoto similarity and the TM-score between the different PDB files used for the docking of organometallic compounds that contain rhenium.

Rhodium

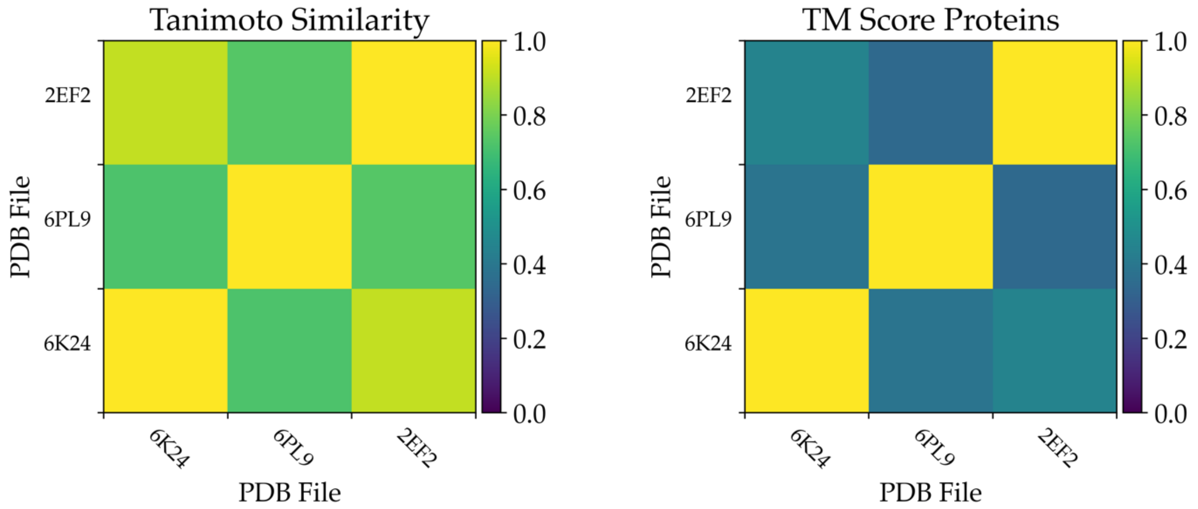

Figure S10. The Tanimoto similarity and the TM-score between the different PDB files used for the docking of organometallic compounds that contain rhodium.

Vanadium

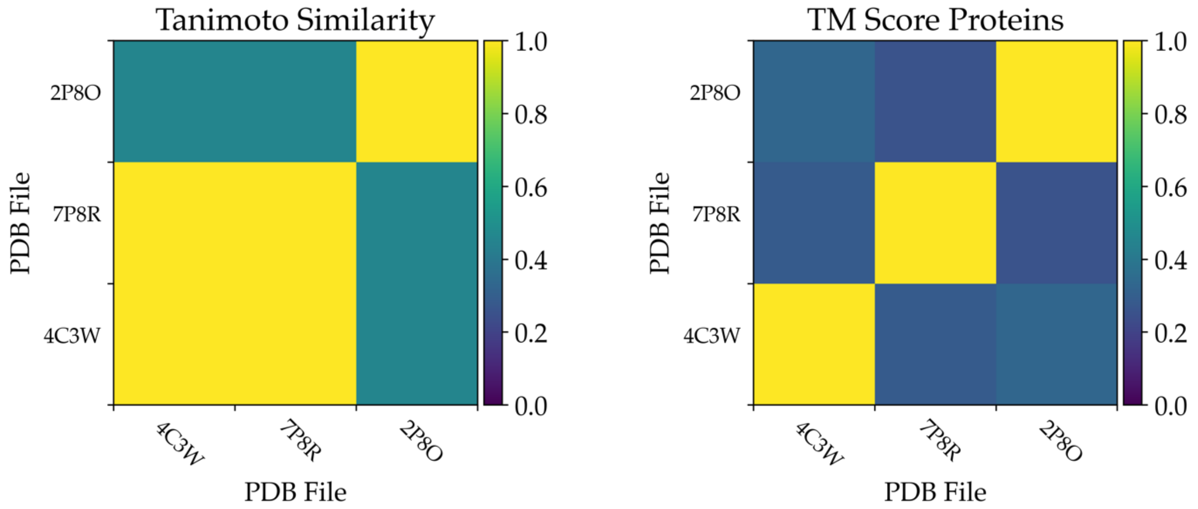

Figure S11. The Tanimoto similarity and the TM-score between the different PDB files used for the docking of organometallic compounds that contain vanadium.
